# One case of sporadic hemiplegic migraine with multiple pulmonary arteriovenous malformation

**DOI:** 10.1007/s10194-011-0292-x

**Published:** 2011-01-19

**Authors:** Mianwang He, Shengyuan Yu, Guangyi Wang

**Affiliations:** General Hospital of PLA, Beijing, China

## Introduction

Hemiplegic migraine is a rare variation of migraine with aura (MA) which should be classified as either familial hemiplegic migraine (FHM) or sporadic hemiplegic migraine (SHM) [1]. SHM is defined as migraine attacks associated with some degree of motor weakness/hemiparesis during the aura phase and no first degree relative (parent, sibling or child) has identical attacks. FHM is the only migraine subtype for which a monogenic mode of inheritance (autosomal dominant) has been clearly established to be caused by mutations in any of the following three gene loci—CACNA1A, ATP1A2 and SCN 1A [2, 3]. Some recent studies have identified involvement of these gene loci in SHM as well [2, 3]. Moreover, SHM is more difficult to diagnose and often requires several investigations to rule out other possible diseases. Friberg et al. [4] studied regional cerebral blood flow (rCBF) in three patients in whom hemiplegic migraine was induced by focal hypoperfusion developed in the frontal lobes and spread posteriorly.

Pulmonary arteriovenous malformation (PAVM) is rare pulmonary vascular anomaly. Although most patients are asymptomatic, PAVM can cause dyspnoea from right-to-left shunt (RLS) and various central nervous system complications from paradoxical emboli [5]. However, there have not been reports on SHM coexisting with RLS from PAVM presented in the case.

## Case

**Fig. 1:**
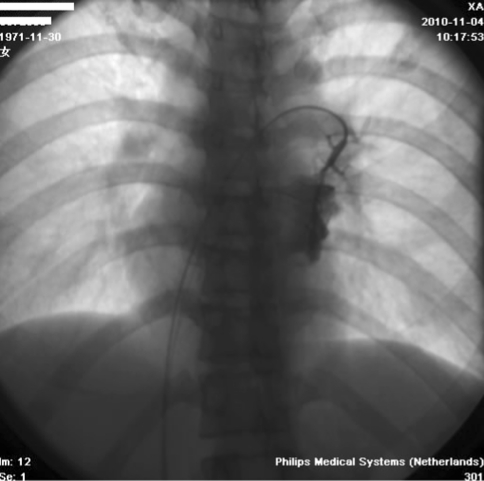
Venous phase of Selective DPSA

A 38-year-old female had episodic headache for 19 years. Prior to headache attack, bright spots or zig-zag line flashes of bilateral visual fields were experienced firstly for about 10 min, then numbness and weakness of right face and of the right side of the body appeared together with motor aphasia that was sometimes preceded by sensory aphasia. All this aura symptoms usually were completely relieved in an hour after onset of visual aura. Half an hour after the onset of visual aura, severe pulsating headache appeared in left fronto-temporal and post-orbital areas, accompanying with nausea, vomiting, photophobia and phonophobia. The patient did not show the disturbance in consciousness, seizure attacks, running nose and conjunctival congestion. Headache symptom usually partly relieved after taking analgesic drugs and sleeping and completely relieved in 3 days. Weakness and tiredness usually persisted for 1–2 days after end of headache attacks. These episodes appeared about 1–2 times per month, usually with tiredness and mood agitation before the attacks. There was not episode for 4 years since 2006. The headache attacks recently relapsed with mood agitation as the prodrome and were more frequent and severe. The patient had headache attack again even when headache symptom of last episode had not completely relieved, and was admitted for further examinations. Otherwise no family history was found. Multiple arteriovenous fistulas with RLS in proximal branch of left lower pulmonary artery were found by trans-esophageal-echocardiography (TEE) and percutaneous digital pulmonary subtraction angiography (DPSA) (Fig. 1), and the diameter of the largest fistula was 1.8 cm, for which the fistula closure was not performed because of multiple fistulas status. No abnormality was found by physical and neurologic examination. No abnormality was found on routine examinations, cerebrolspinal fluid (CSF) and lactate/pyruvate tests, 24 h electroencephalogram (EEG), cranial magnetic resonance imaging (MRI) and magnetic resonance angiography (MRA). During hospitalization period, flunarizine was orally taken 10 mg qn. No headache attack appeared in 2 months except for occasional slightly visual aura with flash spots.

## Discussion

Sporadic hemiplegic migraine was diagnosed according to the (ICHD)-II, based on typical migraine with motor aura and no identical attack in first degree relatives, and excluded other possible reasons. Structural brain lesions such as congenital and vascular abnormalities were excluded by brain imaging studies. The gradual progression symptom and severe prolonged post-ictal headache phase together with normal EEG examination excluded the possibility of transient ischemic attack (TIA) and epilepsy, and the motor weakness and aphasia aura excluded the possibility of basilar migraine. The possibility of mitochondrial encephalopathy could be excluded by normal neuroimaging and normal plasma lactate/pyruvate ratio, and pseudomigraine with lymphocytic pleocytosis was excluded by normal CSF results. It might support the diagnosis that flunarizine was effective. Flunarizine is one of first-line agents for prevention of migraine in EFNS guideline [6] and is effective for hemiplegic migraine [7].

The gradual development and sequential march of visual, sensory, motor and aphasia aura symptom could be explained by cortical spreading depression (CSD) mechanism. CSD is a brief excitation of the occipital cortical neurons, which initiate a depolarization wave that moves across the cortex at a rate of 3–5 mm/min, and is followed by prolonged depression of the neurons [8]. Regional cerebral blood flow was decreased during aura phase of MA which started in the occipital region and spread forward at a rate of 2–3 mm/min sideways causing the migraine aura [9, 10]. In our patient, visual aura firstly appeared and then numbness and weakness of right face and of the right side of the body appeared together with motor aphasia, which could infer that CSD originated from the occipital lobe and then sequentially spread to the frontal, temporal and parietal lobe of left hemisphere.

There might be association between RLS from PAVM and SHM. Brighina et al. (2009) [11] reported a case of SHM coexisting with patent foramen ovale (PFO). However, the role of PFO in migraine pathogenesis remains to be unclear. There is no sufficient evidence that recurrence of migraine is improved by PFO closure based on the recently published migraine intervention with STARFlex Technology (MIST) trial data [12]. Post et al. [13] found that embolization of PAVMs in patients with hereditary hemorrhagic telangiectasia was associated with amelioration of migraine. Post et al. [14] proved the association between MA and PAVM. Na et al. [15] reported a case of symptomatic improvement of migraine headache after surgical resection of the pulmonary lobe with PAVM. But Woods et al. [16] found that small and moderate-size RLS did not appear to be significantly associated with migraine headache. The causal link cannot be established in our case, because there was no way to verify the potential effect of PAVM on SHM because the fistula closure was not performed. At present, the association between MA and RLS is under investigation, and the mechanism is not fully understood.

RLS might have a role in the aetiology of MA and paradoxical gas embolism might precipitate MA [17]. Wilmhurst et al. [18] suggested that migraine attack was associated with a significant RLS which allowed a venous agent, possibly 5-hydroxytryptamine, to bypass the lung filter. Migraine can occur when there is no shunt if similar agents are liberated in the left heart beyond the lung filter, possibly by platelet activation. In this case of SHM coexisting PAVM, RLS showed by TEE and DPSA supports the hypothesis that the RLS promote the occurence of MA including SHM by some venous agents to bypass the filter.

Although the causal link between PAVM and SHM has not been assured, RLS might play a role of pathogenesis of MA including SHM. The question remains to be open.
